# Plant Nitrogen Acquisition Under Low Availability: Regulation of Uptake and Root Architecture

**DOI:** 10.1093/pcp/pcw052

**Published:** 2016-03-29

**Authors:** Takatoshi Kiba, Anne Krapp

**Affiliations:** ^1^RIKEN Center for Sustainable Resource Science, 1-7-22 Suehiro, Tsurumi, Yokohama, 230-0045 Japan; ^2^Institut Jean-Pierre Bourgin, Institut Jean-Pierre Bourgin, INRA, AgroParisTech, CNRS, Université Paris-Saclay, RD10, 78026 Versailles, France

**Keywords:** Acquisition efficiency, Limitation, Nitrogen nutrient, Root architecture, Uptake

## Abstract

Nitrogen availability is a major factor determining plant growth and productivity. Plants acquire nitrogen nutrients from the soil through their roots mostly in the form of ammonium and nitrate. Since these nutrients are scarce in natural soils, plants have evolved adaptive responses to cope with the environment. One of the most important responses is the regulation of nitrogen acquisition efficiency. This review provides an update on the molecular determinants of two major drivers of the nitrogen acquisition efficiency: (i) uptake activity (e.g. high-affinity nitrogen transporters) and (ii) root architecture (e.g. low-nitrogen-availability-specific regulators of primary and lateral root growth). Major emphasis is laid on the regulation of these determinants by nitrogen supply at the transcriptional and post-transcriptional levels, which enables plants to optimize nitrogen acquisition efficiency under low nitrogen availability.

## Introduction

Nitrogen (N) availability is a major factor determining plant growth and productivity. Plants can acquire N through their roots from the soil under inorganic (nitrate and ammonium) and organic (e.g. urea, amino acids, peptides) forms. Although organic forms contribute to plant N nutrition in specific habitats such as in boreal ecosystems (Jones and Kielland 2012, Werdin-Pfisterer et al. 2012), nitrate and ammonium are the universal forms in most soils. In natural soils, their availability is generally low but can also be highly variable depending on various factors including soil physical properties, leaching and microbial activity, which often result in the formation of N depletion areas in the soil (Jackson and Caldwell 1993, Miller and Cramer 2004). To face such low N availability conditions, plants display elaborate responses to enhance N use efficiency (Good et al. 2004, Hermans et al. 2006, Nacry et al. 2013).

N use efficiency has been defined in multiple ways; however, in general, it can be divided into two components, N utilization efficiency and N acquisition (uptake) efficiency. Uptake activity and root architecture are the major determinants of the acquisition efficiency (reviewed in Glass 2003, Garnett et al. 2009, Xu et al. 2012). The former is facilitated by influx transporters located on the plasma membrane, and the latter by alterations in growth and development in response to local and systemic N signals (Forde 2014, Krapp et al. 2014). Needless to say these two factors are co-ordinated to optimize acquisition. This review aims to summarize recent advances in our understanding of the mechanisms that plants employ to increase N acquisition efficiency under low N availability, with special reference to the regulation of uptake and root architecture in *Arabidopsis thaliana* (Arabidopsis). In this review, we mostly focus on ‘N limitation (sudden complete deprivation, low or growth-limiting concentrations)’ and ‘heterogeneous supply (nutrient patches simulated by split-root)’.

## Regulation of Uptake

Ammonium and nitrate are taken up actively into root cells by different sets of plasma membrane-localized transporters. Ammonium transport is mediated by transporters of the AMT/MEP/Rh (AMT) superfamily (Ludewig et al. 2007). Six *AMT* genes exist in Arabidopsis, all of which encode high-affinity ammonium transporters (Loque et al. 2006, Yuan et al. 2007a). For nitrate uptake, two families of transporters, NPF, for NITRATE TRANSPORTER 1/PEPTIDE TRANSPORTER family (previously named the NRT1/PTR family) and NRT2, have been identified (Nacry et al. 2013, Krapp et al. 2014). In Arabidopsis, there are 53 and seven members in the NPF and NRT2 families, respectively. The NPF members studied so far have a low affinity for nitrate (Leran et al. 2014), except AtNPF6.3 (NRT1.1), which has dual-affinity transport and nitrate-sensing functions (Ho et al. 2009). NRT2 members are high-affinity nitrate transporters, and most of them require another component, NAR2 (NRT3), to mediate nitrate transport (Kotur et al. 2012, Gu et al. 2014, Liu et al. 2014). A series of genetic and physiological studies have shown that high-affinity transporters plays a central part in efficient N uptake under low availability (Krapp et al. 2011, Gu et al. 2013).

## Molecular Basis of Ammonium Uptake

Five of the six *AMT* genes in Arabidopsis, namely *AtAMT1;1*, *AtAMT1;2*, *AtAMT1;3*, *AtAMT1;5* and *AtAMT2;1*, are expressed in roots, and their transcript levels are up-regulated under N limitation (Yuan et al. 2007a). Ammonium influx studies using triple and quadruple mutants showed that AtAMT1;1, AtAMT1;2 and AtAMT1;3 are additively responsible for about 90% of high-affinity uptake capacity under N limitation, and that AtAMT1;5 most probably accounts for the remaining capacity (Loque et al. 2006, Yuan et al. 2007a). AtAMT1;1, AtAMT1;3 and AtAMT1;5 are expressed chiefly at the root tip and in epidermal cells, while AtAMT1;2 is localized in the endodermis and cortex. All AtAMTs have different ammonium affinity levels and transport capacities indicative of their roles in planta. AtAMT1;1, AtAMT1;3 and AtAMT1;5 act to absorb ammonium directly from the soil, and AtAMT1;2 transports apoplastic ammonium into the cell (Loque et al. 2006, Yuan et al. 2007a). These lines of evidence illustrate how important it is for the regulation of multiple transporters with appropriate substrate affinity and capacity to be co-ordinated for effective ammonium uptake under low availability.

Although *AMT* expression is derepressed by N limitation in Arabidopsis, ammonium-inducible expression has been reported in other plants such as rice, tomato and maize (Sonoda et al. 2004, Gu et al. 2013). Furthermore, the rice and poplar genomes contain 10 and 14 putative *AMT* genes, respectively (Sonoda et al. 2004, Couturier et al. 2007), suggesting that the co-ordinated regulation patterns of *AMT* genes could be quite intricate depending on plant species and habitat.

## Molecular Basis of Nitrate Uptake

**Fig. 1:**
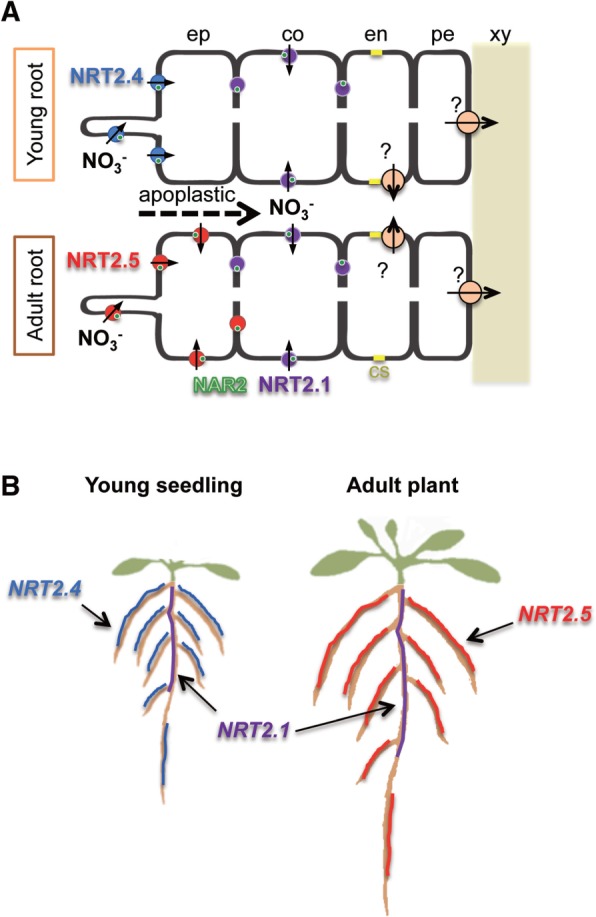
Schematic illustration summarizing the function of NRT2 transporters in Arabidopsis roots under low N availability. Spatial and temporal localization of AtNRT2.1 (NRT2.1, purple), AtNRT2.4 (NRT2.4, blue) and AtNRT2.5 (NRT2.5, red) in (A) root tissues and (B) whole root systems under low N availability. (A) The NRT2.4/NAR2 complex is localized to the outer (soil) side of the epidermal cells of the roots of young seedlings. The NRT2.5/NAR2 complex is expressed in the epidermal cells of the roots of adult plants. NRT2.4 and NRT2.5 are responsible for nitrate uptake directly from the soil. Nitrate can apoplastically penetrate toward cortex cells to be absorbed by the NRT2.1/NAR2 complex. NAR2 (AtNAR2.1) is shown as green circles. Orange circles indicate a putative high-affinity exporter involved in xylem loading of nitrate. (B) NRT2.1 is strongly expressed in the older part of the root system, while NRT2.4 and NRT2.5 are preferentially expressed in the younger part of the roots of young seedlings and adult plants, respectively. ep, epidermis; co, cortex; en, endodermis; pe, pericycle; xy, xylem; cs, casparian strip

Among seven *NRT2* genes in Arabidopsis, *AtNRT2.1*, *AtNRT2.2*, *AtNRT2.4* and *AtNRT2.5* are expressed in the roots of N-deprived plants. Analysis of a quadruple mutant revealed that these four NRT2 transporters account for approximately 95% of high-affinity nitrate influx activity under N limitation, AtNRT2.1 being the major contributor (Lezhneva et al. 2014). Recent studies suggest that the spatio-temporal distribution of these four AtNRT2 transporters is critical for efficient nitrate uptake to sustain growth under low N availability (Fig. 1; Kiba et al. 2012, Lezhneva et al. 2014). During N deprivation, the expression of *AtNRT2.1* is transiently derepressed in the cortex cells of older parts of primary and lateral roots (Wirth et al. 2007). In contrast, the transcript levels of *AtNRT2.4* and *AtNRT2.5* increase during N deprivation over time in the epidermal cells of young primary and lateral roots (Kiba et al. 2012, Lezhneva et al. 2014, Kotur and Glass 2015). These spatial expression patterns indicate that AtNRT2.4 and AtNRT2.5 are responsible for nitrate uptake from the soil, and AtNRT2.1 plays a role in apoplastic nitrate absorption. Although *AtNRT2.4* and *AtNRT2.5* are expressed in the same cell types, the former is predominant in young seedlings and the latter in adult plants (Fig. 1; Kiba et al. 2012, Lezhneva et al. 2014). In addition, AtNRT2.4 was suggested to have much higher affinity for nitrate than AtNRT2.1 (Kiba et al. 2012). Although a dual-affinity transporter AtNPF6.3 is also expressed in roots under N limitation, its direct contribution to high-affinity nitrate transport under N limitation seems to be minor, maybe even non-existent (Glass and Kotur 2013). The existence of a high-affinity efflux transport system for xylem loading of nitrate (Fig. 1A) has been suggested from the phenotype of *atnrt1.5*, which is a mutant of a low-affinity efflux transporter responsible for xylem loading of nitrate (Lin et al. 2008). However, the transporter gene(s) involved in the system is(are) still unknown.

The NRT2 family genes have also been investigated in other plant species, and many N-limitation-inducible genes have been identified (Guo et al. 2014, Pellizzaro et al. 2015). Biochemically, they seem to act as high-affinity nitrate transporters (Yan et al. 2011, Gu et al. 2014). However, functional characterization of these genes in planta remains to be carried out.

## Regulation of High-Affinity N Transporter Genes at the Transcript Level

**Fig. 2:**
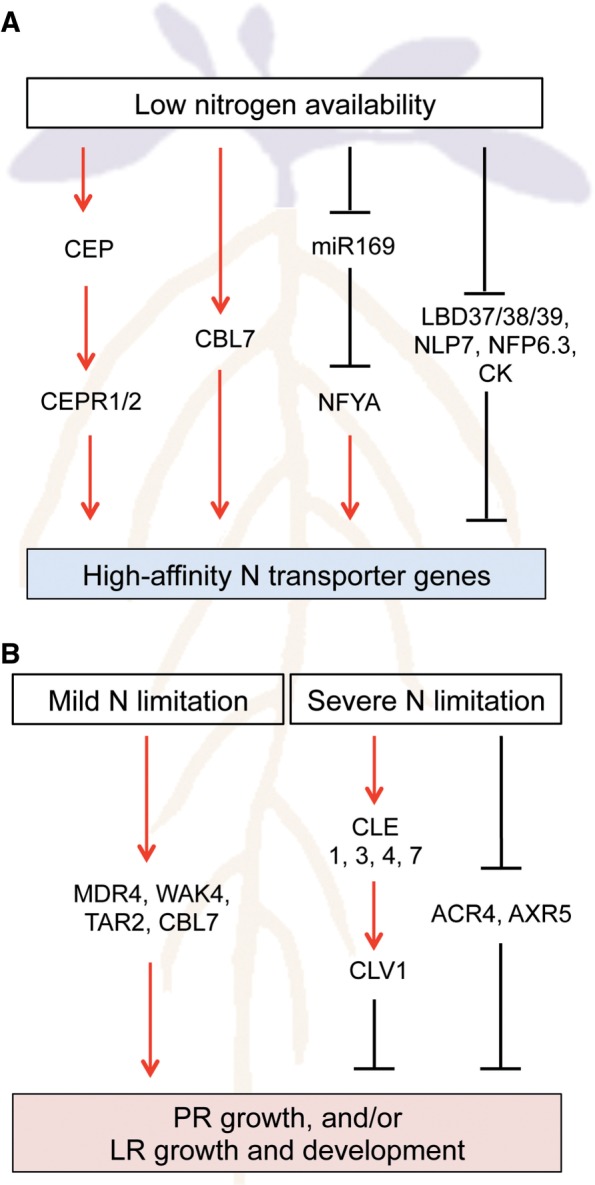
A model of low N availability signaling pathways involved in the regulation of high-affinity N transporter gene expression and root architecture in Arabidopsis. Signaling pathways regulating (A) the expression of high-affinity N transporter genes (*AtNRT2* and *AtAMT* genes) and (B) primary root (PR) growth, and/or lateral root (LR) growth and development under low N availability are depicted. Only pathways described in this review are shown. Red arrows and black blunted lines indicate positive and negative interactions, respectively. CK, cytokinin

Generally the expression of genes encoding high-affinity transporters of mineral nutrients is induced (or derepressed) under low substrate availability. This is also the case for all *AtAMT1* and *AtNRT2* genes involved in uptake, as described above. A reduction of the internal pool of glutamine and/or derived metabolites is thought to be one of the signals for induction (Lejay et al. 1999, Rawat et al. 1999, Yuan et al. 2007a, Nacry et al. 2013). Recently *AtNPF6.3* was shown to be involved in the negative regulation of induction under high N availability. In the knockout mutant of *AtNPF6.3* (*chl1-5*) grown under high N availability, the expression of high-affinity N transporter genes including *AtNRT2.1*, *AtNRT2.4* and *AtAMT1;3* was derepressed (Fig. 2A; Munos et al. 2004, Bouguyon et al. 2015). Phosphorylation of the threonine (T) 101 residue (T101) plays a role in this regulation. Introducing the phosphomimetic mutant form AtNPF6.3^T101D^ into *chl1-5* restored repression, but the non-phosphorylatable mutant form AtNPF6.3^T101A^ did not. However, how low N availability is sensed and how the signal is transduced through phosphorylation of AtNPF6.3 is still unknown.

LATERAL ORGAN BOUNDARY DOMAIN (LBD) family transcription factors (LBD37/38/39) and members of the RWP-RK family transcription factor NIN-LIKE PROTEIN (NLP) are implicated in the regulation of high-affinity N transporter genes under low N availability in Arabidopsis. Overexpression of *LBD37/38/39* suppressed some N limitation responses, including the induction of *AtNRT2.1, AtNRT2.2* and *AtNRT2.5* (Fig. 2A). The expression of *LBD37/38/39* is low under N limitation, while it is high under N sufficiency, indicating that LBD37/38/39 function as repressors of N limitation responses under N sufficiency (Rubin et al. 2009). Recently NLP6 and NLP7 were shown to act as master regulators of the primary nitrate response in Arabidopsis (Castaings et al. 2009, Konishi and Yanagisawa 2013, Marchive et al. 2013). Interestingly, *nlp7* mutants display constitutive N limitation responses, including induction of *AtAMT1;5* and *AtNRT2.5* (Castaings et al. 2009), indicating that NLP7 also plays a role in the repression of N limitation responses. Indeed, chromatin immunoprecipitation analysis revealed that NLP7 is bound to high-affinity N transporter genes such as *AtAMT1;1*, *AtNRT2.1* and *AtNRT2.5*, showing direct regulation (Fig. 2A; Marchive et al. 2013).

The expression of high-affinity N transporter genes is also regulated by systemic N signals. Cytokinins, a class of plant hormones, have been proposed to act as both local and systemic signals co-ordinating N demand and acquisition (Kiba et al. 2011). A positive correlation between tissue and vascular cytokinin contents and N availability has been reported in many plant species (Hirose et al. 2008, Kiba et al. 2011, Kamada-Nobusada et al. 2013). Exogenous application of cytokinins represses *AtNRT2* genes in N-deprived roots (Kiba et al. 2011), suggesting that cytokinins act as N sufficiency signals to suppress nitrate uptake by the roots (Fig. 2A). Consistently, split-root experiments showed that cytokinins are involved in systemic N deficiency (demand) signaling to regulate *AtNRT2.1*, *AtNAR2* and *AtNRT2.4* under heterologous N supply (Ruffel et al. 2011).

MicroRNAs (miRNAs) are also implicated in local and systemic N signaling. Although a number of miRNAs responsive to low N availability have been identified in various plant species (Pant et al. 2009, Liang et al. 2015, Nguyen et al. 2015), few of them have been characterized. Arabidopsis miR169, which targets *NUCLEAR FACTOR Y, SUBUNIT A* (*NFYA*) family members, is so far the only one shown be involved in the regulation of N uptake under low N availability. miR169 is down-regulated by N limitation, while the transcript levels of *NFYA* genes are up-regulated (Pant et al. 2009, Zhao et al. 2011). Transgenic plants overexpressing *MIR169a* display reduced levels of *NFYA* transcripts and show hypersensitivity to N limitation, which is associated with decreased *AtNRT2.1* expression (Zhao et al. 2011). The promoter region of *AtNRT2.1* contains an NFY-binding consensus sequence, suggesting that miR169 regulates *AtNRT2.1* through NFY (Fig. 2A). Interestingly, miR169 was abundantly detected in rapeseed phloem sap (Pant et al. 2009), implying its role as a systemic N signal. Recently Tabata et al. (2014) identified C-TERMINALLY ENCODED PEPTIDEs (CEPs) as systemic N deficiency (demand) signals under heterologous N supply in Arabidopsis. CEPs are small peptides produced in the N-deprived side of the root. After translocation to the shoot, CEPs are perceived by two leucine-rich repeat receptor kinases CEP RECEPTOR1/2 (CEPR1/2) that increase *AtNRT2.1* and *AtNPF6.3* expression and nitrate uptake in the other side of the root (Fig. 2A; Tabata et al. 2014). However, the mechanism whereby shoot-located CEPR1/2 activates *AtNRT2.1* and *AtNPF6.3* expression in the root remains to be elucidated.

Stability of the mRNA encoding high-affinity N transporter genes can also regulate efficient uptake. Transcript stability of *AtAMT1;1* is regulated by N availability (Yuan et al. 2007b). The *AtAMT1;1* transcript level driven by the *Cauliflower mosaic virus 35S* RNA promoter accumulated under N limitation in tobacco, while its level decreased after resupply of ammonium or nitrate.

## Regulation of High-Affinity N Transporters at Post-Translational Levels

Post-translational regulation of transporters would be of benefit because it enables plants to respond immediately to sudden changes in N availability. The best characterized transporters for post-translational regulation include AtAMT1;1 and AtAMT1;3. The transport activity of AtAMT1;1 and AtAMT1;3 is regulated by the phosphorylation status of a threonine residue in the cytosolic C-terminal domain. Phosphorylation, which represses transport activity, occurs when plants are grown with ammonium, but does not occur during N limitation (Yuan et al. 2013). AtNPF6.3 is also regulated by phosphorylation in response to nitrate availability. AtNPF6.3 is phosphorylated at T101 by CBL-INTERACTING PROTEIN KINASE 23 (CIPK23) under low nitrate to function as a high-affinity transporter, while it is dephosphorylated under high nitrate to be a low-affinity transporter (Liu and Tsay 2003, Ho et al. 2009). Crystallography suggested that dimer decoupling caused by T101 phosphorylation is relevant to the change in affinity for nitrate (Parker and Newstead 2014, Sun et al. 2014).

Several studies suggest that AtNRT2s are also regulated post-translationally. When *AtNRT2.1* was expressed under the control of the 35S promoter, the activity of high-affinity transporters was repressed by high N supply, even though mRNAs accumulated constantly. Under the same conditions, AtNRT2.1 and AtNAR2.1 protein levels were not correlated with the activity (Wirth et al. 2007, Laugier et al. 2012), implying post-translational regulation. Regulation could involve dissociation of the AtNRT2.1/AtNAR2.1 complex (Yong et al. 2010), cleavage of the C-terminus (Wirth et al. 2007) and phosphorylation of AtNRT2.1 (Engelsberger and Schulze 2012), though the relevance of such regulation to nitrate uptake under low N availability remains to be demonstrated.

The polar localization of transporters is believed to be important for efficient nutrient uptake. Transporters of various micronutrients, such as the boron transporters AtBOR1 and AtNIP5;1, the silicon transporters OsLsi1 and OsLsi2, a manganese transporter OsNRAMP5, and an iron transporter AtIRT1 display lateral polarity (Miwa and Fujiwara 2010, Barberon et al. 2014, Ma and Yamaji 2015). However, reports of polar localization of macronutrient transporters are scarce. Among N transporters, AtNRT2.4 is so far the only one shown to have a polar localization. Its localization to the outer (soil) side of epidermal cells is likely to be important for AtNRT2.4 to operate with much higher affinity than AtNRT2.1 (Fig. 1A; Kiba et al. 2012). Although the molecular mechanism of AtNRT2.4 polar localization is unknown, endocytic trafficking between the plasma membrane and endosomes may be a common underlying mechanism for the establishment of polarity for mineral transporters (Takano et al. 2010, Barberon et al. 2014). Consistently, single-particle fluorescence imaging revealed that AtAMT1;3 on the plasma membrane is regulated by clustering and endocytosis under high ammonium (Wang et al. 2013).

## Regulation of Root Architecture Under Low N Availability

Plants can improve nutrient uptake by modulating root growth and architecture. By increasing the total absorptive surface of the root system and directing growth toward nutrient-rich patches of the soil, plants are able to adapt to nutrient availability in the soil. Taking into account the high mobility of nitrate in the soil and the restricted amount of nitrate available within a given soil area, the definition of the most efficient root architecture might not be that obvious and may vary depending on plant species, soil type and other environmental parameters (Postma et al. 2014). Modeling approaches suggest that efficient nitrate capture results from a trade-off between the speed of N acquisition and the total volume of soil explored (Dunbabin et al. 2003).

Several different aspects of N-dependent modulation of root morphology are reported and discussed in recent reviews (Forde 2014, Giehl et al. 2014). The nutritional status of the plant as well as local signals detected by the roots trigger morphological changes in the overall root system. Nitrate, ammonium and glutamate act locally on the roots and induce different morphological responses (Walch-Liu and Forde 2008, Lima et al. 2010). The modification of root growth under N limitation depends on the strength of N limitation and on other environmental conditions, such as light intensity and day length. Primary and lateral root length is increased under mild N limitation (Lopez-Bucio et al. 2003, Gruber et al. 2013), whereas total root development is delayed under severe N limitation, leading to short primary roots and a proportionally reduced number of lateral roots (Araya et al. 2015).

A survey of the expression of about 100 root development-related genes in the publicly available transcriptome data of Arabidopsis (Giehl et al. 2014) revealed that N limitation induces the expression of the WALL ASSOCIATED KINASE 4 (WAK4) and of the shootward auxin transporter MULTIDRUG RESISTANCE 4/P-GLYCOPROTEIN 4 (MDR4/PGP4). Both genes stimulate primary and lateral root growth (Lally et al. 2001, Terasaka et al. 2005) and might be involved in the response to mild N limitation (Fig. 2B). On the other hand, down-regulation of the expression of *ARABIDOPSIS CRINKLY 4* (*ACR4*) and *AUXIN RESISTANT 5* (*AXR5*), both involved in lateral root formation (Yang et al. 2004, De Smet et al. 2008), might indicate that these genes are involved in the reduction of lateral root formation under severe N limitation (Fig. 2B).

The regulatory mechanisms involved in the responses to changing N availability are multiple, and suggest a complex regulatory network also involving hormonal regulation. In particular, auxin plays a determining role not only in the local nitrate response (Krouk et al. 2010), but also in the response to N limitation in Arabidopsis (Ma et al. 2014). Under mild N limitation, auxin accumulates in the non-emerged lateral root primordia with more than three cell layers, leading to increased lateral root growth. This is accompanied by an increase in the expression of the auxin biosynthesis gene *TRYPTOPHAN AMINOTRANSFERASE RELATED 2* (*TAR2*) in the pericycle and the vasculature of the mature root zone near the root tip. Loss of function of TAR2 impairs auxin accumulation and lateral root growth. Conversely, overexpression of *TAR2* increases lateral root numbers under both high and low N supply. This causal correlation between TAR2 and auxin accumulation and lateral root number is thus part of the regulation of root architecture under low N availability (Fig. 2B).

Also based on the up-regulation of gene expression under N limitation, members of the *CLAVATA3/ESR-RELATED* (*CLE*) gene family are part of a (probably) different regulatory mechanism that restrictively controls the expansion of the lateral root system in N-limited environments in Arabidopsis (Araya et al. 2014). Expression of peptides CLE1, 3, 4 and 7 is induced under N limitation chiefly in the root pericycle cells. Their overexpression leads to reduced lateral root growth. CLE peptides are ligands of the CLAVATA1 (CLV1) leucine-rich repeat receptor-like kinases. CLV1, the receptor of CLE3, is expressed in phloem companion cells. The overexpression of CLE3 in a *clv1* mutant background did not modify root architecture. In addition, the *clv1* mutant was impaired for N-regulated lateral root primordia outgrowth. This signaling module is localized in the root vasculature, and acts under severe N limitation as a mechanism to prevent the expansion of the lateral root system into N-poor environments (Fig. 2B).

A link with calcium signaling exists, as the CALCINEURIN B-LIKE PROTEIN 7 (CBL7) is involved in the regulation of root growth upon nitrate limitation in Arabidopsis (Ma et al. 2015). *cbl7* mutants display reduced primary root growth specifically under very low N availability (Fig. 2B). This growth phenotype is accompanied by a decreased root nitrate content and reduced expression of *AtNRT2.4* and *AtNRT2.5.* The corresponding regulatory mechanism still needs to be further revealed. However, calcium (Ca^2+^) has recently been shown to act as a secondary messenger in the primary nitrate response in Arabidopsis (Riveras et al. 2015).

## Perspectives

Because of the environmental and economic impacts of excessive fertilizer use, there is a growing demand for new crop varieties suited for low-input sustainable agriculture (Mueller et al. 2012). Considering the importance of N, improving N acquisition efficiency, whatever the breeding technique may be, could be one promising approach to generate such crops. Thus it is imperative to identify the mechanisms that plants inherently possess to enhance N acquisition under low N availability. As summarized in this review, there has been a great deal of progress in our understanding of the genes and signaling pathways that regulate N uptake and root architecture (Figs. 1, 2). Although high-affinity N transporter genes have been thoroughly characterized in Arabidopsis, it is still unclear how N acquisition could be improved by manipulating high-affinity N transporters. Several attempts have been made to improve N acquisition efficiency by constitutively expressing a high-affinity transporter gene, without much success (Fraisier et al. 2000, Kumar et al. 2006, Katayama et al. 2009, Bao et al. 2015). Since it has become evident that spatio-temporal orchestration of multiple transporters is a key mechanism underlying efficient uptake of N (Fig. 1) and of other nutrients (Miwa and Fujiwara 2010, Ma and Yamaji 2015), co-ordinated manipulation of multiple transporters may be an effective strategy to improve N acquisition. Thus, it will be of great interest to investigate the mechanisms whereby this orchestration is achieved.

Positive coincidences between quantitative trait loci (QTLs) for N uptake and root architecture have been observed in maize (Coque et al. 2008, Garnett et al. 2009), suggesting that optimal root architecture would improve N acquisition. However, our understanding of molecular components that regulate the root architecture in response to N availability is mostly limited to Arabidopsis. Whether the function of the components is equivalent in other plants, especially in monocots, needs to be tested. Furthermore, one future challenge will be to understand how the transporter orchestration and the modulation of root architecture are co-ordinated to maximize acquisition efficiency in a fluctuating environment.

## Funding

This work was supported by the Japan Society for the Promotion of Science [a Grant-in-Aid for Young Scientists (A) (No. 26712009 to T.K.)]; the National Agency for research (ANR) [grant ANR-14-CE19-0008 to A.K.]; LabEx Saclay Plant Sciences-SPS [for work in the laboratory of A.K. (ANR-10-LABX-0040-SPS)].

## Disclosures

The authors have no conflicts of interest to declare.

## Abbreviations

AMT: ammonium transporter
CBL: calcineurin B-like protein
NRT: nitrate transporter
